# Teachers’ Perceptions of Technology Integration in Teaching-Learning Practices: A Systematic Review

**DOI:** 10.3389/fpsyg.2022.920317

**Published:** 2022-06-06

**Authors:** Huma Akram, Abbas Hussein Abdelrady, Ahmad Samed Al-Adwan, Muhammad Ramzan

**Affiliations:** ^1^Department of Education, Northeast Normal University, Changchun, China; ^2^Department of English Language and Translation, Qassim University, Buraydah, Saudi Arabia; ^3^Department of Electronic Business and Commerce, Al-Ahliyya Amman University, Amman, Jordan; ^4^Department of English Linguistics, The Islamia University of Bahawalpur, Bahawalpur, Pakistan

**Keywords:** technology integration, teachers’ perceptions, teaching-learning practices, online teaching challenges, online learning, systematic review

## Abstract

The rapid expansion of the Information and Communication Technologies (ICTs) has transformed learners into digital learners, requiring teachers to integrate technology into their pedagogical approaches, where teachers’ attitudes, technological knowledge, and skills play a significant role in its effective integration. From this perspective, the current study presents teachers’ perceptions regarding technology integration in their teaching-learning practices at all educational levels in light of the previous studies performed in the last 5 years in Pakistan. The findings reveal that teachers exhibit positive perceptions regarding technology integration in teaching-learning practices. They believe that technology-incorporated teaching assists them in enhancing their instructional practices effectively, making the learning process exciting and interactive, and keeping learners motivated. Regarding barriers, the slow speed of the internet, load shedding, lack of infrastructure, online teaching experience, and training were reported as the main obstacles that hinder teachers from effective integration of ICT into their teaching practices. Accordingly, the study findings suggest that concerned authorities should set clear and effective policies to make efficient use of ICT by allocating a sufficient budget and ensuring all necessary facilitation (e.g., ICT infrastructure, tools, software, internet, and labs) in all educational institutions. Furthermore, particular attention should be devoted to supplying adequate opportunities for the career development of teachers in developing technological competencies, which help them successfully use ICT in their instructional practices.

## Introduction

Being an essential part of the present time, Information and communication technology (ICT) significantly influences all domains of human life (Gnambs, 2021). Similarly, ICT has also transformed the education sector and turned instructional practices into more interactive and productive (Lin et al., 2017), as it offers various tools which are used in traditional as well as online teaching spaces and assists in building a proactive classroom environment (Jogezai et al., 2021). Technology-incorporated instructional practices not only enhance the quality of teaching (Akram et al., 2021a) but also enable students to develop their skills, boost their motivation, and enhance their knowledge and information efficiently (Chen et al., 2018). During the COVID-19 global crisis, when the entire world’s activities across all domains of human lives got restricted, ICT played a supporting role in sustaining teaching-learning activities on the one hand (Thaheem et al., 2021). While on the other hand, ICT-integrated teaching and learning provided a flexible approach and better access to learning opportunities as a substitute for face-to-face instruction (Akram et al., 2021b). However, teachers faced difficulties in making the best use of ICT in their instructional practices due to inadequate technological competencies, yet the transitory phase improved their digital skills. Furthermore, the utilization of ICT in education for enhancing instructional practices’ effectiveness has been considered crucial for the last few decades worldwide (U.S. Department of Education, 2017). Several studies also highlighted the significance of ICT-integrated instructional approaches in meeting the educational needs of the learners by increasing their thoughtfulness and keeping students motivated, which is viewed as a significant predictor of students’ educational growth (Xu et al., 2021). Liu et al. (2022) also identified that technology-integrated learning increases the cognitive understanding and learning achievements of students. In addition, ICT incorporated teaching-learning practices also enable learners to stay connected with their instructors and peers (*via* various social media platforms), help students resolve their academic challenges and keep them participating actively in the learning activities (Liu Z. et al., 2021). Recognizing the importance of students’ active involvement in the learning activities, Liu S. et al. (2021) also suggested teachers design collaborative activities *via* the computer-supported collaborative concept mapping (CSCCM) technique to develop a collaborative classroom environment, which also enhances students’ interest. By putting it briefly, ICT integrated teaching-learning is the requirement of time, which allows the learners to satisfy their learning needs and helps teachers align their teaching approaches with global standards.

Correspondingly, Pakistan has also recognized the importance of ICT in education, and the national educational policies have proved that the government has special concerns about integrating ICT into the teaching-learning practices to meet the global needs (Pakistan Ministry of Education, 2018). However, there are many factors in developing countries, including Pakistan, such as; lack of ICT infrastructure (Akram et al., 2021a), electricity and internet (Akram et al., 2021b), technological knowledge and expertise (Asad et al., 2020), and lack of teacher training in educational institutions (Abbasi et al., 2021), that badly affects the successful usage of ICT in classrooms. Apart from above mentioned technology-related factors, teachers’ personal perceptions and beliefs play an essential role in effective technology integration. As their beliefs define their pedagogical decisions on how to integrate technology within their instructional practices that support the teaching and learning needs of the twenty first century (Tondeur et al., 2017). Previous studies have also identified that teachers’ instructional practices are greatly affected by their pedagogical beliefs (Taimalu and Luik, 2019). Teachers prefer those technological applications that align with their pedagogical strategies and existing beliefs about teaching and learning practices. In other words, technology use is greatly associated with teachers’ perspectives regarding the nature of teaching and learning in a classroom. Taking this into account, innovative educational strategies suggest that technology integration can only be comprehended well when teachers’ perspectives regarding technology use are taken into consideration (Watson and Rockinson-Szapkiw, 2021). In this regard, this study was intended to investigate teachers’ perceptions regarding advantages, their willingness, attitudes, and challenges they encounter while integrating ICT in their teaching-learning practices. By knowing teachers’ experiences and perceptions, this study would help teachers make the best use of ICT in teaching and learning activities by sorting out the challenges and assisting concerned authorities in formulating policies accordingly.

## Methodology

The central objective of this systematic analysis was to present a succinct account of representative literature dealing with the teachers’ perceptions and experiences while integrating ICT in their teaching-learning practices. The best literature review provides a full reflection, which helps develop theory, indicate needed areas, and put them in a nutshell (Petticrew and Roberts, 2008). By considering the fact that a literature review entails an organized and invariable approach (Littell et al., 2008), the researcher followed all the tactics of the method. It started with the introduction and then determined the selection criteria of databases, timing, and search terms; subsequently, the selection and classification of the articles are described, which will portray the full picture of the systematic approach. Lastly, before proceeding toward the discussion and conclusion, the study’s limitations are conferred.

The employed systematic review approach entails the, which entails the selection, evaluation, and analysis of reports and findings of the prior studies, which subsequently allows a researcher to come to clear conclusions regarding the subject unidentified by the previous researchers. Originally, this method was initially used for medical studies, but Denyer and Tranfield (2009) identified that this approach may also be applied in management sciences studies. Later on, a systematic review was declared as a standard approach for the location, assortment, and assessment of research that may assist policymakers, researchers, and academia in utilizing those findings. According to Denyer and Tranfield (2009), the systematic review technique entails these five phases:

Phase-1: structuring questions/objectives
Phase-2: Finding prior studies
Phase-3: Selecting and evaluating prior studies
Phase-4: Analyzing and synthesizing prior findings
Phase-5: Using and reporting the results.

Among these five phases, the first phase was depicted in the introduction, whereas the subsequent stages were discussed in the methodology and the result sections. Furthermore, to increase the transparency of the findings, each step of the systematic review was explained precisely (Saunders et al., 2012).

### Databases Selection

**FIGURE 1:**
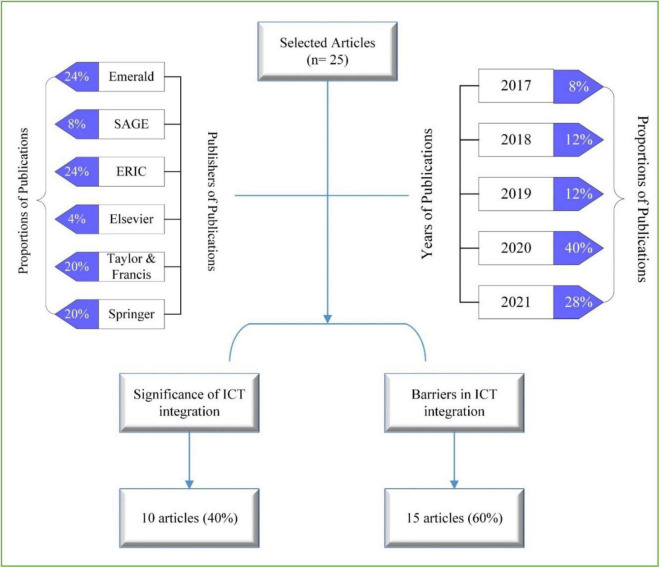
Thematic classification and studied articles by years and publishers.

The study adopted the approach of Denyer and Tranfield (2009) to explore teachers’ experiences, perceived challenges, and emerging topics regarding the integration of ICT in teaching-learning practices. Subsequently, the search across all major subscribed publishers/platforms was conducted, such as Emerald, Springer, SAGE, Taylor & Francis, Elsevier, and ERIC (Figure 1), and articles were taken from the reputed peer-reviewed academic journals to increase the authenticity of the findings (Saunders et al., 2012).

Keeping in view to report the updated literature, the articles from the last 5 years between the time-span of 2017–2021 were selected (Figure 1). In this inquiry, 25 publications were included that were selected by specifying the different types of search terms, such as teachers’ perceptions, beliefs, usage, and attitudes toward ICT integration, and encountered challenges in their application. These key terms were further searched by combining them with terms like school and higher-level to acquire a wide range of information. Furthermore, to investigate the integration of ICT in teaching practices, the terms such as integration, usage, incorporation, and adoption were used to acquire more specific results. All the selected articles were checked manually by checking their abstracts to make sure the articles fell within the scope of the study. The papers out of the scope were excluded based on the publication time, location, and domain, and finally, 25 articles were selected.

### Data Analysis

The data from the selected articles was analyzed by means of content analysis, which is a rigorous approach used to analyze prior documents such as official reports, books, and publications. This approach is commonly known as an efficient way to reduce the number of sources and qualitatively analyze the features of the documents (Onwuegbuzie et al., 2012). Teachers’ perceptions of technology integration in teaching-learning practices were analyzed by thoughtfully reading the literature and paying special attention to identifying the factors contributing to the successful integration of ICT in their instructional practices. Subsequently, the results of the reviewed studies were tabulated separately using a Microsoft Word document and coded on the basis of their respected information. All the factors were then classified into two main groups (Figure 1), i.e., benefits and barriers perceived by teachers during the integration of ICT in their educational practices.

### Limitation of Research

Every study includes certain limitations due to a number of constraints faced by the researcher. Similarly, this study was also gone through some limitations. Primarily, to gain authentic insights for the systematic review analysis, only peer-reviewed articles from reputed publishers were included. Secondly, the key terms used to search publications involve some limitations; for instance, key terms were searched by combining with specific terms such as primary, secondary, and higher education teachers to explore a wide range of research articles.

## Results

**FIGURE 2:**
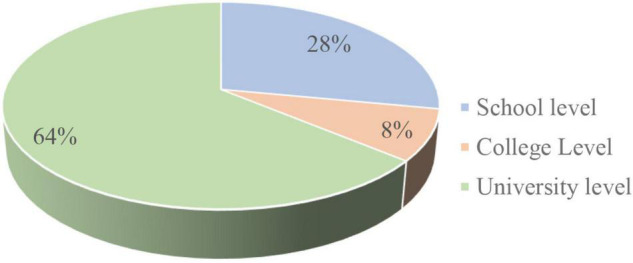
Distribution of articles by educational levels.

The identified information of the outlined articles was found in line with the purpose of this study, which was later sorted chronologically. Keeping in view to identify the integration of ICT in teaching-learning practices at the educational level, the articles were sorted out across different educational levels, i.e., from the school to the university level (shown in Figure 2). Most of the identified studies were conducted at the university level, i.e., 64%, followed by the studies conducted at school (28%) and college (8%) levels, respectively.

**FIGURE 3:**
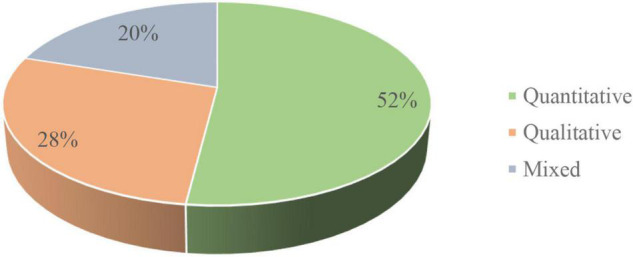
Distribution of articles by research methods.

The nature of research articles with regards to their adopted methodologies contributes significantly to conducting good literature reviews. Hereby, the sampled studies were sorted out across their mode of methodological approaches, which is shown in Figure 3. It signified that the most commonly used methodological approach was the quantitative method, i.e., 52%, followed by the qualitative method (28%), while the least applied approach was the mixed-method approach in the proportion of 20% share.

Teachers’ perceptions of the integration of technology were classified into two paradigms to illustrate the significance and barriers of ICT in their teaching-learning practices. These are described below:

### Significance of Information and Communication Technology Integration

**FIGURE 4:**
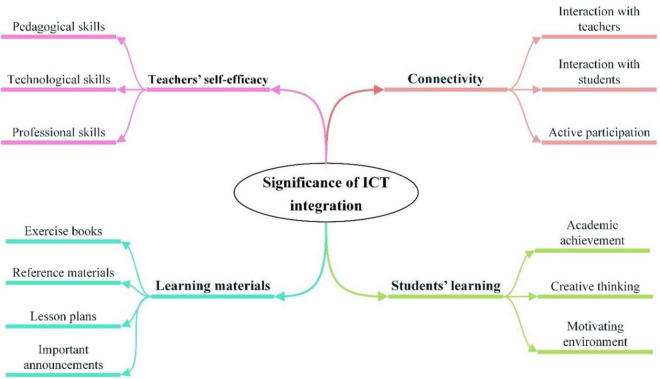
Significance of information and communication technology (ICT) integration.

However, the practical employability of technology in teaching-learning practices is yet to be achieved in Pakistan. Prior studies have highlighted several benefits of integrating ICT into our education system (see Figure 4), which are characterized below.

### Connectivity

According to the majority of the studies, the utilization of ICT for teaching-learning purposes enables teachers and students to stay connected and facilitates learners irrespective of their location and time (Hussain, 2018; Thaheem et al., 2021). Teachers also feel comfortable guiding and discussing with their students within or outside the universities through several digital platforms such as WhatsApp, Facebook, and google groups (Hodgson and Shah, 2017). Regarding the connection of learners with other students, ICT integration in educational practice encourages students to interact with their classmates more (Asad et al., 2020), which helps resolve their academic challenges and keeps them socially active.

Regarding the benefits of ICT integration at the secondary school level, Jogezai et al. (2018) signified that the teachers who use ICT in their instructional practices observed a significant improvement in students’ participation in their learning activities.

### Teachers’ Self-Efficacy

Based on the self-efficacy perspective, effective integration of ICT in education highly depends upon teachers’ self-efficacy (Guoyan et al., 2021), as self-efficacy is a vital force that enables a teacher to acquire students’ learning outcomes successfully. Considering the importance of teachers’ self-efficacy in an effective ICT integration, several studies have suggested developing their pedagogical and technological skills by establishing training programs. The study by Khan and Abid (2021) revealed that the paradigm shift from face-to-face to online teaching-learning during the COVID-19 phase gave an advantage for teachers and students to acquire technological skills by using different digital tools and platforms. They also advocated employing an online certification decision matrix (OCDM) to assure online teaching effectiveness and readiness of teachers.

Furthermore, the findings by Aslam et al. (2021) revealed that ICT integration enhances the quality of the teaching-learning process and found a significant correlation between teachers’ technological pedagogical and content knowledge (TPACK) and their technological competencies. In addition, Abbasi et al. (2021) identified positive attitudes of teachers toward applying technology in their instructional practices and identified a significant association between technology use with their technological competencies.

### Students’ Learning

Teacher plays an important role in cultivating successful students’ online learning. ICT utilization in teaching-learning practices enables a student to make meaningful use of technologies in education by accessing, selecting, establishing, and interpreting the information. Its effective integration help in meeting the learners’ educational needs by providing creative solutions to different types of learning inquiries (Rafi et al., 2019). Several studies also showed a significant relationship between technology usage in educational practices and students’ academic achievement (Asif et al., 2020; Abbasi et al., 2021). Furthermore, to enhance students’ creative thinking and academic performance, Ali et al. (2018) advocated that cloud computing facilitates students’ learning efficiently with better adaptability in a cost-effective way. As adoption of cloud services help educational institutions to fulfill students’ learning needs without having all resources, such as multimedia equipment, software, and hardware. In addition, the application of ICT in classrooms provides a motivating environment for students and keeps them engaged in educational activities (Jogezai et al., 2018).

Furthermore, the disruptive phase of COVID-19 brought an opportunity to enhance the effectiveness of teaching-learning practices through ICT integration at the school level. During the COVID-19, when face-to-face learning opportunities were interrupted due to schools’ closure, the integration of ICT *via* social media platforms helped learners to continue their learning activities (Jogezai et al., 2021).

### Learning Materials

Students’ learning is supported when they receive adequate supplementary materials, such as reference books, exercise books, or teaching aids. The correct use of such materials not only assists them in making their prospective concepts clear but also boosts their academic achievement. In this regard, technology-assisted learning enables a learner to acquire supportive learning materials easily (Hodgson and Shah, 2017). The study by Habib et al. (2021), demonstrated the benefits of a learning management system (LMS) and management information system (MIS), in supporting the administrative, teaching, and learning activities at the university level. For instance, LMS enables teachers to share course outlines, reference materials, lesson plans, assignment submissions, important announcements, and assessment reports. Similarly, LMS also aids students in getting easy access to all reference materials, important announcements, and other relevant information irrespective of their location and time.

Regarding the benefits of technology use at the secondary school level, Asif et al. (2020) signified that the designed and prearranged resources (in the forms of videos or texts) shared by teachers enable the learners to apprehend the lesson clearly before the teacher’s demonstration.

### Barriers in Information and Communication Technology Integration

**FIGURE 5:**
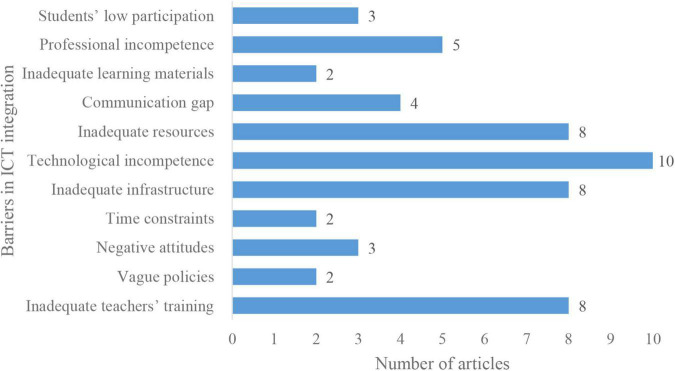
Barriers in information and communication technology (ICT) integration.

The application of technology in educational practices is not a new phenomenon in developed countries. However, its applicability is still not a common practice across developing nations like Pakistan. In this regard, teachers’ perceptions regarding barriers to technology integration in their teaching practices were seen in multiple studies (see in Figure 5).

However, the prior studies conclude that most teachers exhibit expectant perceptions regarding ICT incorporation as a pedagogical tool in their teaching practices. The absence of predefined educational policies has been reported as a big hurdle that prevents teachers from efficiently using technology in their instructional practices. Consequently, the integration of ICT from secondary to higher education levels is uncertain (Afridi and Chaudhry, 2019). However, the government of Pakistan recognizes the importance of ICT in education and introduced several policies to integrate technology into education. Nevertheless, the implementation of those policies has not been seen yet, due to several barriers, which include inadequate teachers’ training and infrastructure, technological incompetence, and accessibility of resources (Dar et al., 2018), poor communication between teacher and student (Shah et al., 2020), inadequate digital competences of students (Rafi et al., 2019), inadequate learning materials (Abbasi et al., 2021), no harmonious instructional design (Ajmal et al., 2019). As a consequence of these barriers, Pakistan is still lagging behind other countries that have adopted technology efficiently in their education system. To overcome these barriers, concerned educational authorities should efficiently formulate effective policies to incorporate ICT in teaching-learning practices and allocate a sufficient budget to attain the policies’ objectives.

Some studies reported low competencies and avoiding the behavior of teachers in applying technology in their instructional practices. For instance, Shah et al. (2020) explored that few teachers reported the fruitful results of adopting ICT in their teaching practices, while other teachers could not find time and feasibility to apply ICT in their teaching practices due to the limited time and tight working schedule.

Amid the COVID-19 crisis, Thaheem et al. (2021) carried out a survey to explore the benefits and challenges confronted by teachers in their online instructional practices, where technological challenges, personal constraints, and lack of infrastructure were reported as the major challenges. Similar challenges have been informed by Soomro et al. (2020) and Noor et al. (2020), where teachers showed resistance to integrating ICT in their instructional practices due to the lack of adequate infrastructure and technological competencies. This situation points out attention for concerned authorities to endow all educational institutions with sufficient and contemporary technologies to acquire optimal students’ learning outcomes.

In addition, teachers’ attitudes are considered a crucial determinant that reflects their behaviors in applying technologies in their pedagogical practices by responding positively or negatively based on their experiences (Abbasi et al., 2021). Concerning ICT application in pedagogical practices, teachers’ attitudes and their professional knowledge were described and examined in multiple studies. For instance, Ahmed et al. (2017) found that several teachers view social interaction between students and teachers important, but they don’t find online teaching interactive compared to face-to-face teaching. Hereby, those teachers prefer to deliver lessons face-to-face and show a negative attitude toward the online teaching mode. At the same time, Afridi and Chaudhry (2019) also found an unsatisfactory status of adopting technologies in instructional practices in all universities of Punjab due to several constraints. The study, therefore, suggests providing adequate assistance to teachers regarding information technology solutions to enable teachers to adopt the latest technologies efficiently.

Online teaching-learning is different from the traditional ones in several aspects, and teachers encounter challenges in facilitating learners appropriately if they are not competent enough. In this regard, teachers’ professional knowledge has been considered a crucial determinant for the effective integration of technology in strengthening their instructional practices (Aslam et al., 2020). However, the study by Asad et al. (2020) described positive responses by the teachers. Still, they don’t find support from their governing bodies to apply ICT in their pedagogical approaches due to the lack of resources and professional competencies. Abbasi et al. (2021) and Thaheem et al. (2021) also indicated that teachers are often found improvised to integrate digital instructional approaches into their curricula effectively. In this regard, concerned educational authorities should provide adequate assistance to teachers to enhance their technological and professional competencies.

Regarding technology use at the secondary school level, it is still found at the initial stages (Jogezai et al., 2020). However, the COVID-19 phase allowed the integration of technologies in teaching-learning at the school level, but its actual integration is yet to be achieved. For instance, Ullah and Ali (2021) found that amid COVID-19, students from elite private schools in the urban areas received an online learning advantage over the students from public schools in the rural areas due to the lack of adequate infrastructure and competent teachers.

## Discussion

The present age of the classroom requires the best teachers who can bring innovation in their instructional practices. In pursuing so, technology plays a significant role in bringing innovation to teaching practices, and its effective integration help students meet their learning needs. Since teachers’ instructional practices are greatly defined by their pedagogical beliefs and prefer those technological applications that align with their pedagogical strategies and existing beliefs about teaching and learning practices. In this regard, the current study gives a deep insight to understand teachers’ perceptions regarding advantages, their willingness, attitudes, and challenges they encounter while integrating ICT into their teaching-learning practices. The analysis disclosed a considerable expansion of the number of articles in relation to the technology integrated teaching-learning practices over the last 5 years. Regarding educational level, most of the identified studies were conducted at the university level, followed by the school and college levels, respectively. In conformity with this finding, Turan and Akdag-Cimen (2020) also observed that technology integrated educational research remained widespread among university-level participants due to easy access and to follow the trend. However, integration of technology in teaching-learning practices is believed to be beneficial in meeting learners’ needs at all levels. In this regard, more studies should be conducted at college and school levels to gain valuable insight regarding technology integrated teaching-learning practices.

However, Pakistan has not achieved the desired implementation of technology-incorporated teaching-learning practices yet. The reviewed articles have revealed various bright sides of integrating ICT into our educational practices. The most consistently informed positive feature is students’ academic growth which can be attributed to a motivating environment that keeps them engaged in active learning activities. Furthermore, technology-assisted learning also enables a learner to acquire supportive learning materials easily, which helps in making their prospective concepts clear and boosts their academic achievement. This finding reflects the findings of Liu et al. (2022), where learners reported a high level of cognitive understanding and learning achievements in the MOOC discussion forum. Thus, in order to enhance students’ learning achievements, it is considered worthwhile for teachers to frame inquiry-based and open discussion activities integrated with the learning materials.

Another reported benefit from the reviewed studies is that ICT incorporated teaching-learning practices enable teachers and students to stay connected and help students resolve their academic challenges. Liu Z. et al. (2021) also advocated the use of social media platforms to support learners to participate in online learning activities actively. Therefore, teachers should assign various communicative and group discussion tasks to learners to optimize their communication with their instructors and classmates to enhance their understanding and resolve certain issues.

Furthermore, teachers’ professional and technological competencies play a key role in integrating ICT in their instructional practices effectively, which greatly depends upon their self-efficacy. The reviewed articles of the study also confirm that ICT usage in teaching and learning practices boosts teachers’ competencies in several aspects. Watson and Rockinson-Szapkiw (2021) also pointed out that technology-integrated instructional practices not only enhance the quality of teaching but also boost teachers’ pedagogical and technological skills. In contrast to the benefits, the technological incompetence of teachers emerged as the most frequently reported challenge in light of the reviewed studies. A similar challenge has been identified by Hassan (2021), where faculty members encountered several difficulties in adopting ICT in their instructional practices due to inadequate technological competencies. It is, therefore, essential for concerned authorities to play a supportive role by organizing training programs to strengthen technological skills among teachers. Besides, the government should take special concerns to ensure the successful integration of ICT in educational institutions and provide some incentives or certificates to teachers to encourage their efforts.

Adequate resources and updated infrastructure play a key role in the effective integration of technology in educational practices. In contrast, inadequate infrastructure and limited resources emerged as the second-largest reported challenges from the reviewed studies that hinder teachers from effective technology integration in instructional practices. García-Morales et al. (2021) also reported a similar challenge and advocated that adequate infrastructure and technological resources are vital to sustain effective teaching and learning practices. In this regard, concerned authorities should allocate a sufficient budget to facilitate teachers and students by providing adequate resources and updated infrastructure to make the best use of ICT in educational practices.

In addition, students’ active involvement with an apparent communication with their teachers and classmates is essential for their efficient learning. On the other hand, reviewed articles inform that teachers don’t find online teaching more interactive than face-to-face teaching due to the lack of apparent communication with students and their active participation. It is, therefore, essential for teachers to design computer-supported collaborative concept mapping (CSCCM) activities in the classroom to strengthen students’ understanding, which would help them enhance the level of collaboration with their teachers and peers (Liu S. et al., 2021).

Recognizing the needs of the time and setting educational goals and objectives accordingly is the core responsibility of governing bodies to acquire the desired outcomes (Akram and Yang, 2021). In contrast, the absence of predefined educational policies has been reported as a big hurdle in the reviewed articles that hinders educational practices from incorporating technology efficiently and become difficult to overcome the barriers. Thus, to eliminate all obstacles, concerned educational authorities should efficiently formulate effective policies to incorporate ICT in teaching-learning practices that meet the country’s current needs and academic situations at all levels.

Adequate time plays a significant role in fulfilling the teaching responsibilities of teachers required to incorporate ICT in their instructional practices efficiently. In contrast, the reviewed articles inform that teachers don’t find adequate time to perform all the pedagogical tasks required to integrate ICT in their educational practices, such as lesson planning, making notes, or lectures. A similar barrier has been identified by Akram et al. (2021b), where teachers reported that they don’t find enough time to make the efficient use of ICT in raising their instructional practices’ effectiveness. Therefore, it is essential to facilitate teachers by providing professional development and time management programs, either pre-service or in-service teachers, to combat the prospective constraints.

## Conclusion

The systematic review affirms that teachers exhibit adequate acceptance of ICT in the teaching-learning practices and assist learners in acquiring learning objectives in several ways, which is why inevitable at all educational levels. However, technology use at the secondary school level is still found at the initial stages than at the higher level. Still, the changes brought by the COVID-19 pandemic brought an opportunity to enhance the effectiveness of teaching-learning practices through ICT integration at all levels. Findings also specified several barriers that hinder effective technology integration in teaching-learning practices, including lack of resources, leadership support, accessibility of ICT infrastructure, inadequate time, unclear policies, professional development, technical support, and lack of appropriate pedagogical models. In this context, concerned authorities should work on the needs and gaps which hinder educational practices from effective technology integration to acquire maximum benefits from technology-integrated teaching and learning.

## Author Contributions

HA was the principal investigator of the study and conducted from conceptualization to the data analysis. AHA, ASA, and MR refined the study and helped in the revision.

## Conflict of Interest

The authors declare that the research was conducted in the absence of any commercial or financial relationships that could be construed as a potential conflict of interest.

## Publisher’s Note

All claims expressed in this article are solely those of the authors and do not necessarily represent those of their affiliated organizations, or those of the publisher, the editors and the reviewers. Any product that may be evaluated in this article, or claim that may be made by its manufacturer, is not guaranteed or endorsed by the publisher.
